# Preoperative Intravitreal Conbercept Facilitates Vitrectomy in Proliferative Diabetic Retinopathy: Is Attention Required for the Fellow Eye?

**DOI:** 10.1155/2019/2923950

**Published:** 2019-08-05

**Authors:** Wei Xu, Weijing Cheng, Yao Yao, Jian Guo, Guoxing Xu

**Affiliations:** ^1^Department of Ophthalmology, The First Affiliated Hospital of Fujian Medical University, Fuzhou, China; ^2^Fujian Institute of Ophthalmology, Fuzhou, China

## Abstract

**Purpose:**

To evaluate the effect of preoperative intravitreal conbercept (IVC) injection on patients with proliferative diabetic retinopathy (PDR).

**Methods:**

Medical records of patients who underwent vitrectomy due to complications of PDR were retrospectively reviewed. Patients were grouped as the IVC group and non-IVC group according to preoperative IVC. Preoperative, intraoperative, and postoperative data of both eyes were collected. The main outcome measures were best-corrected visual acuity (BCVA), intraocular pressure (IOP), central retinal thickness (CRT), and incidence of tractional retinal detachment (TRD).

**Results:**

A total of 37 cases were included, 16 in the IVC group and 21 in the non-IVC group. Preoperative IVC significantly reduced surgical duration (IVC vs. non-IVC, 88.9 ± 11.9 min vs. 97.8 ± 12.8 min, *p*  <  0.05). In the vitrectomized eye, no significant difference existed between the IVC group and non-IVC group regarding postoperative BCVA (logMAR, 1.20 ± 0.53 vs. 1.27 ± 0.54, *p* = 0.68), IOP (16.5 ± 2.9 mmHg vs. 15.6 ± 3.7 mmHg, *p* = 0.44), and CRT (330.1 ± 35.2 *μ*m vs. 319.2 ± 32.5 *μ*m, *p*=0.34). In the fellow eye, 6 cases in the IVC group were diagnosed with TRD during postoperative follow-up, while only 2 cases were diagnosed in the non-IVC group (*p*  <  0.05).

**Conclusion:**

Preoperative intravitreal injection of conbercept has effectively facilitated vitrectomy in PDR patients, but it potentially promotes tractional retinal detachment in the fellow eye following preoperative injection.

## 1. Introduction

Diabetic retinopathy (DR) is one of the main reasons that results in vision loss in developed countries around the world [1]. Although progress in understanding the pathogenesis of diabetes mellitus (DM) has lead to significant reduction of its complications including DR, the prevalence of any DR was about 35% in people with DM [2]. Among those people with DR, a substantial proportion of patients progress into the proliferative stage when the disease is not controlled properly. Proliferative diabetic retinopathy (PDR) is a sight-threatening condition for DR patients and brings a significant source of social burden.

At present, there are few measures to prevent DR except active treatment of DM. However, emerging therapeutic strategies against DR in the recent decades have effectively deterred progressive visual impairment to the patients. The application of panretinal photocoagulation (PRP) prior to severe PDR-related complications, e.g., vitreous hemorrhage and tractional retinal detachment, has reduced severe vision loss to almost 50% [3]. Continuous improvement of surgical technique in vitrectomy also allows nonclearing vitreous hemorrhage and tractional retinal detachment that result from PDR being treated. The recent advent of anti-vascular endothelial growth factor (VEGF) antibodies has revolutionized the treatment of diabetic macular edema. Preoperative administration of anti-VEGF drugs facilitates surgical procedure for PDR, although the long-term outcome remains under investigation.

Conbercept (Kanghong Inc., Chengdu, China) is a recombinant fusion protein that primarily targets VEGF receptors. Intravitreal injection of conbercept in PDR patients before or immediately after vitrectomy was reported to significantly improve surgical outcomes regarding intra- and postoperative complications and visual outcomes [4, 5]. The adjunctive administration of conbercept in PDR patients improves the PRP completion rate and patient satisfaction [6]. Anti-VEGF therapy brings advantage in the management of PDR while attention should be paid to potential progression of vitreoretinal fibrosis [7]. Although avoidance of vitreoretinal proliferation in the injected eye can be achieved by timing of preoperative injection, little was known to the fellow eye. In this study, we reviewed outcomes of both eyes in PDR patients who underwent preoperative conbercept injection and compared them with those in patients without preoperative conbercept.

## 2. Materials and Methods

This retrospective study was approved by the Ethics Board of The First Affiliated Hospital of Fujian Medical University and complied with the tenets of the Declaration of Helsinki. Written informed consent was obtained from all subjects. Medical records of patients who underwent vitrectomy to treat nonclearing vitreous hemorrhage or tractional retinal detachment with macula-off due to PDR at The First Affiliated Hospital of Fujian Medical University between September 2015 and August 2018 and had at least 6 months of postoperative follow-up were reviewed. All patients were fully informed regarding the potential benefits and risks of preoperative intravitreal conbercept (IVC) injection to make independent decisions. Patients were grouped into IVC group and non-IVC group according to preoperative IVC.

Fellow eye in this study was defined as the nonvitrectomized eye upon case inclusion. The inclusion criteria were as follows: The glycosylated hemoglobin (HbA1c) was controlled below 10% for at least 3 months. Patients had no intraocular surgery in both eyes within the last 6 months. No intravitreal injection was previously performed in both eyes. A color Doppler image revealed vitreoretinal proliferation before vitrectomy. Exclusion criteria included: opacity of cornea or lens in the fellow eye, tractional retinal detachment in the fellow eye, intravitreal injection in the fellow eye during follow-up, history of ocular trauma, diagnosed with glaucoma, hypertension with oral angiotensin-converting enzyme inhibitor (ACEI), or angiotensin receptor blocker (ARB).

Preoperative data collection included age, gender, duration of DM, type of DM, HbAc1, best-corrected visual acuity (BCVA), and PDR grading of the fellow eye. Patients underwent phacoemulsification combined with intraocular lens implantation. Subsequently, 23-gauge pars plana vitrectomy was performed, and fibrovascular proliferative membranes were removed with necessary endodiathermy. Perfluorodecalin (DK-Line®, Bausch & Lomb, Rochester, USA) was injected to reattach the retina for PRP. The liquid was cleared via air/fluid exchange, and retinal tamponade was achieved with silicone oil (Oxane® 5700, Bausch & Lomb, Rochester, USA). The intraoperative data were collected as surgical duration and incidence of iatrogenic retinal hole. The postoperative outcome measures were BCVA, intraocular pressure (IOP), central retinal thickness (CRT), and incidence of tractional retinal detachment in the fellow eye.

Statistical analysis was performed by SPSS software, version 17.0 (SPSS, Inc., Chicago, IL, USA). All measurement data were presented as mean ± SD, and the differences between the IVC group and non-IVC group were assessed by *t*-test. Differences in categorical variables were assessed by the chi-square test. A *p* value of less than 0.05 was considered to indicate statistical significance.

## 3. Results

A total of 37 cases were included in this study. Preoperative IVC ranged from 5–10 days before vitrectomy. Demographic data of the patients are shown in Table 1. The average age of the patients who underwent vitrectomy due to different complications of PDR was 50.1 ± 10.1 years in the IVC group in contrast with 51.9 ± 8.3 years in the non-IVC group. Gender ratios (male/female) were 7/9 in the IVC group and 10/11 in the non-IVC group. The mean duration of DM in patients from the IVC group and non-IVC group was 8.8 ± 5.0 years and 7.0 ± 4.0 years, respectively. Type 2 diabetes mellitus (T2DM) consisted of the main proportion of PDR patients that required vitrectomy in either IVC group or non-IVC group. No significant difference existed between the IVC group and non-IVC group regarding the preoperative HbAc1. Preoperative BCVA was converted to logMAR scale, and the results were collected. BCVA of the vitrectomized eyes in the IVC group in contrast with the non-IVC group was 2.14 ± 0.28 versus 2.27 ± 0.86, while that of the fellow eyes was 0.72 ± 0.38 versus 0.79 ± 0.55. Statistical significance existed in neither vitrectomized eyes nor fellow eyes regarding preoperative BCVA between the IVC group and non-IVC group. Indication for vitrectomy was nonclearing vitreous hemorrhage or tractional retinal detachment with macula-off. However, 6 cases in the IVC group and 15 cases in the non-IVC group were diagnosed with both nonclearing vitreous hemorrhage and tractional retinal detachment with macula-off.

Postoperative follow-up lasted for 6 months. In vitrectomized eyes, the IOP at the last visit was 16.5 ± 2.9 mmHg in the IVC group versus 15.6 ± 3.7 mmHg in the non-IVC group without significant difference (*p*=0.44). Although preoperative conbercept reduced intraoperative complications (Table 2), postoperative BCVA of the IVC group versus non-IVC group showed no statistical difference at the last visit (1.20 ± 0.53 versus 1.27 ± 0.54, *p*=0.68). Central retinal thicknesses of the vitrectomized eyes at the last visit were 330.1 ± 35.2 *μ*m in the IVC group and 319.2 ± 32.5 *μ*m in the non-IVC group. Also, no significant difference was identified (*p*=0.34). In the fellow eye, data were also collected during postoperative follow-up. Independent-samples *t*-test did not reveal a significant difference between the IVC group and non-IVC group regarding BCVA of the fellow eyes at the last visit (0.71 ± 0.22 versus 0.78 ± 0.35, *p*=0.48), nor did significant difference exist between the IVC group and non-IVC group regarding postoperative IOP at the last visit (15.3 ± 2.1 mmHg versus 14.7 ± 2.7 mmHg, *p*=0.48). Interestingly, 6 cases of tractional retinal detachment were detected in the fellow eyes of those patients who underwent preoperative administration of conbercept in the contralateral eye, while only 2 cases of tractional retinal detachment in the fellow eye were detected in patients without preoperative conbercept (*p* < 0.05, chi-square test). The PDR grades and PRP completion rates in the fellow eyes were thereafter collected, since they may affect progression into tractional retinal detachment. However, the data revealed no significant difference in PDR grading (*p*=0.89, chi-square test) or in PRP completion (*p*=0.79, chi-square test) between the IVC group and non-IVC group (Table 3).

**Figure 1:**
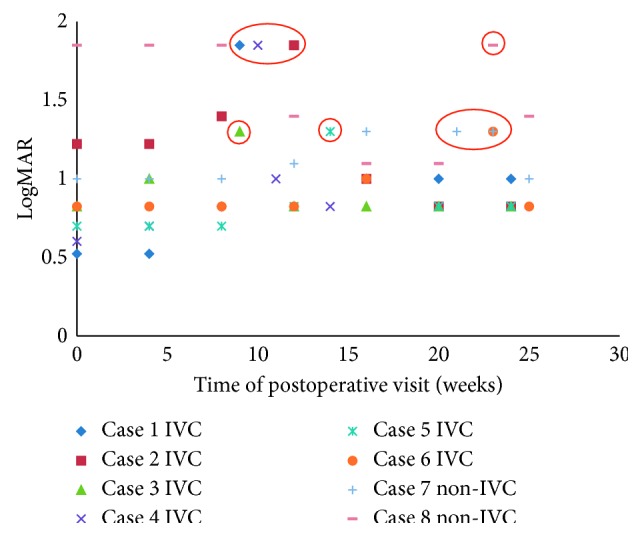
BCVA of the fellow eye during postoperative follow-up. Decreased BCVA occurred in 5 cases (all from the IVC group) around 3 months (from 9 weeks to 14 weeks), the time when tractional retinal detachment was diagnosed (red circles). Three cases were diagnosed with tractional retinal detachment at 21 weeks and 23 weeks following surgery in the contralateral eye, but BCVA decreased only in 2 cases.

**Figure 2:**
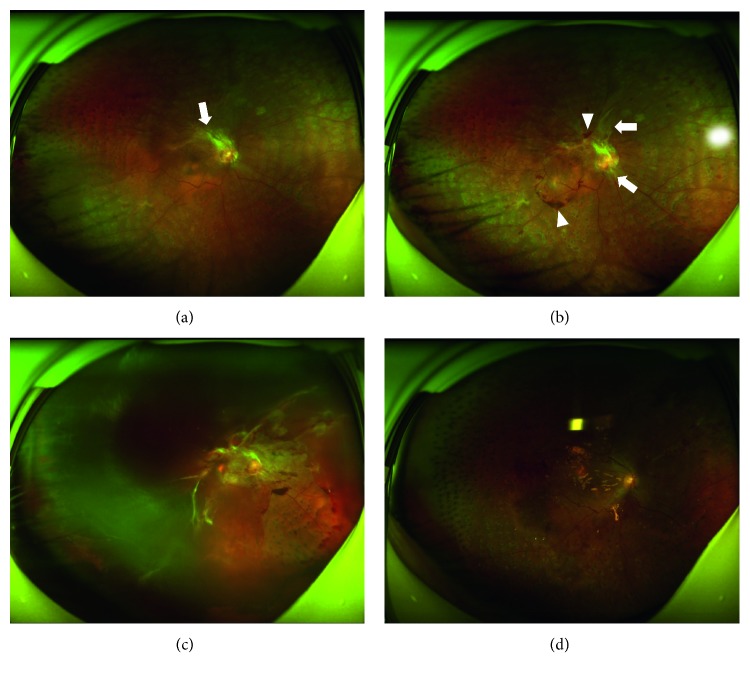
Representative case of progressive proliferation in the fellow eye. Mild proliferation (arrow) appeared in the superior vascular arch before intravitreal conbercept injection in the contralateral eye (a). However, progressive proliferation (arrows) and hemorrhage (arrowheads) subsequently occurred 4 weeks thereafter (b). Tractional retinal detachment and vitreous hemorrhage were diagnosed 9 weeks later (c). Although the retina was reattached after vitrectomy in the fellow eye, visual outcome was poor (d).

Among those patients with tractional retinal detachment in the fellow eyes during postoperative follow-up, the time of occurrence ranged from 4 weeks (1 month) to 23 weeks (5 months). In the IVC group, 5 cases progressed into tractional retinal detachment in the fellow eye around 3 months (from 9 weeks to 14 weeks, Figure 1). One case progressed into tractional retinal detachment in the fellow eye at 23 weeks (5 months after operation). By contrast, the two cases in the non-IVC group were diagnosed with tractional retinal detachment in the fellow eye at 21 weeks and 23 weeks (5 months after operation). Upon diagnosis of tractional retinal detachment, the patients underwent vitrectomy in the fellow eye (Figure 2). Details of cases that were identified with tractional retinal detachment in the fellow eye are shown in Table 4.

## 4. Discussion

Preoperative anti-VEGF provides a new approach in management of intra- and postoperative PDR-related complications. The administration of intravitreal bevacizumab 5–10 days in PDR patients before vitrectomy was reported to significantly reduce intra- and postoperative complications, e.g., iatrogenic retinal breaks and postoperative vitreous hemorrhage, as well as shorten surgical duration. In this study, preoperative conbercept was found to facilitate surgical process for PDR patients, although visual outcomes had no significant difference during postoperative follow-up. In addition, we found progression of PDR in the fellow eye, as observed that incidence of tractional retinal detachment was higher in the fellow eye of patients who underwent preoperative conbercept in the contralateral eye. Similar effect was reported in preoperative administration of bevacizumab in PDR patients [8]. The mechanism inside this phenomenon is obscure, but several aspects may contribute to the progressed proliferation in the fellow eye.

The molecular structure of conbercept makes it highly bioactive in anti-VEGF. The affinity of conbercept to VEGF exceeds even that to the native VEGF receptor [9]. However, this molecular structure may also contribute to potential side effects, as we found that proliferation progressed in the fellow eye. Conbercept is a recombinant fusion protein with a molecular weight of 143 kDa. It acts as a soluble decoy receptor that binds all isoforms of VEGF-A, VEGF-B, and placental growth factor [10], which is similar to aflibercept (115 kDa). The structure of conbercept differs from that of aflibercept in that the fourth Ig domain of VEGFR2 enables conbercept a high affinity to VEGF165 by enhancing the association rate of VEGF [11], even though domain 4 does not participate in ligand binding. The introduction of domain 4 also prolongs half-life of the drug. The half-life of conbercept in the vitreous was estimated to be 4.2 days in rabbits, which is almost two times longer than that of ranibizumab. Although serum concentrations of conbercept become undetectable within 1-2 days after intravitreal injection during phase I trial, serum obtained from patients with choroidal neovascularization or polypoidal choroidal vasculopathy during the follow-up period after intravitreal injection of conbercept revealed that serum concentrations of VEGF significantly reduced, lasted for 1 week, and regressed at 1 month [12]. It suggested that serum conbercept may still be effective even though it is undetectable. Unilateral injection of conbercept may achieve bilateral effect. Interestingly, reduced serum VEGF induced progression of PDR later in the fellow eye instead. Whether it is rebound proliferation following clearance of serum conbercept in the fellow eye or not requires further investigations. However, the animal model test revealed that serum VEGF concentrations rebounded to higher levels than baseline after decreasing for a short time [13]. The elevated serum VEGF perhaps contributes to progression of PDR in the fellow eye, while proliferation in the contralateral eye is not evident due to enough laser coagulation in the surgery and silicone oil tamponade.

Aqueous and choroidal circulation is considered to be the route for clearance of anti-VEGF drugs from the vitreous [14, 15]. The route also allows the drug to be introduced into systemic circulation. Although the vitreous was removed by vitrectomy together with conbercept dissolved inside it, intraocular manipulations and fluctuation of IOP during surgery may cause breakdown of the blood-retina barrier. This in turn promoted conbercept entering bloodstream, especially in patients with longer surgical duration. In addition, vitrectomy created a vitreous with lower viscosity that allowed for easier dispersion of drug residuals as well as clearance from the vitreous cavity and into the systemic circulation compared to eyes without vitrectomy [16]. The concentration of conbercept may be effective to neovascularization at even as low as 1% of normal dose [17]. Therefore, conbercept that reaches the fellow eye through systemic circulation is able to impact the process of PDR, despite the extent of contribution is unknown. Another factor that may contribute to systemic dispersion of anti-VEGF drug is lensectomy. It is reported that serum levels of intravitreal bevacizumab were significantly elevated in eyes following lensectomy [18]. In our study, lensectomy was performed in all cases, accounting for increased release of conbercept into circulation. The elevated serum anti-VEGF drug may potentially improve PDR in the fellow eye, but later clearance of conbercept from serum will result in proliferation instead. Our results revealed that progression of proliferation mainly occurred around 3 months after operation.

The administration of preoperative anti-VEGF drugs in PDR has effectively reduced surgery-related complications and surgical duration by regression of retinal neovascularization and decrease of intraoperative hemorrhage. However, attentions were hardly paid to the potential side effects. Our findings suggested the importance of early preventive intervention to the fellow eye before preoperative anti-VEGF to the contralateral eye since later progression of proliferation was observed following preoperative conbercept. It is therefore necessary to make overall assessment on the fellow eye when taking preoperative anti-VEGF in the contralateral eye. Preventive laser photocoagulation on the fellow eye could be considered for those patients with high risk of progression of proliferation early before preoperative anti-VEGF, but the effectiveness requires evaluation. Since progression of proliferation was still found in case with photocoagulation in this study.

The limitations of our results lie in that selection of preoperative conbercept was decided by the patient after informed consent, making it a nonrandomized study. Their decisions could be influenced by financial considerations. Hence, patients with more complicated physical condition might probably refuse preoperative conbercept. The data from patients without preoperative conbercept seem complex and severe despite no statistical significance. Also, further concerns regarding prognostic factors of proliferation in the fellow eye following preoperative anti-VEGF in the contralateral eye are necessary.

## 5. Conclusions

Preoperative intravitreal injection of conbercept has effectively facilitated vitrectomy in PDR patients, but it is possible to progress proliferation in the fellow eye following preoperative injection. Our results revealed the significance of overall assessment and early intervention to the fellow eye before preoperative anti-VEGF in PDR patients.

## Figures and Tables

**Table 1:** Demographic data and preoperative details of patients.

	IVC group	Non-IVC group
Age (years)	50.1 ± 10.1	51.9 ± 8.3
Gender (cases)		
Male	7	10
Female	9	11
Duration of DM (years)	8.8 ± 5.0	7.0 ± 4.0
Type of DM (cases)		
T1DM	3	2
T2DM	13	19
HbAc1 (%)	7.79 ± 0.91	7.31 ± 0.77
BCVA (logMAR)		
VE	2.14 ± 0.28	2.27 ± 0.86
FE	0.72 ± 0.38	0.79 ± 0.55
Indication for vitrectomy (cases)		
Nonclearing VH	4	3
TRD with macula-off	6	3
Mixed	6	15

*Note*. DM, diabetes mellitus; T1DM, type 1 diabetes mellitus; T2DM, type 2 diabetes mellitus; HbAc1, glycosylated hemoglobin; BCVA, best-corrected visual acuity; VE, vitrectomized eye; FE, fellow eye; VH, vitreous hemorrhage; TRD, tractional retinal detachment.

**Table 2:** Surgical details of the vitrectomized eye and data of both eyes during follow-up.

	Surgical duration (min)	Iatrogenic hole (cases)	BCVA (logMAR)	IOP (mmHg)	CRT (*μ*m)	TRD (cases)
VE						
IVC	88.9 ± 11.9^*∗*^	4	1.20 ± 0.53	16.5 ± 2.9	330.1 ± 35.2	N/A
Non-IVC	97.8 ± 12.8	6	1.27 ± 0.54	15.6 ± 3.7	319.2 ± 32.5	N/A
FE						
IVC	N/A	N/A	0.71 ± 0.22	15.3 ± 2.1	N/A	6^*∗*^
Non-IVC	N/A	N/A	0.78 ± 0.35	14.7 ± 2.7	N/A	2

*Note*. BCVA, best-corrected visual acuity; IOP, intraocular pressure; CRT, central retinal thickness; TRD, tractional retinal detachment; VE, vitrectomized eye; FE, fellow eye; N/A, not applicable. ^*∗*^*p* < 0.05.

**Table 3:** PDR grading and PRP completion in the fellow eye.

	IVC group	Non-IVC group
PDR grading (cases)		
Non-high-risk	5	7
High-risk	11	14
PRP completion (cases)		
Completed	10	14
Not completed	6	7

*Note*. PDR, proliferative diabetic retinopathy; PRP, panretinal photocoagulation. High-risk PDR was defined according to the EDTRS criteria.

**Table 4:** Details of cases that progressed into tractional retinal detachment in the fellow eye.

	Case 1	Case 2	Case 3	Case 4	Case 5	Case 6	Case 7	Case 8
Age (years)	49	63	53	33	35	49	45	69
Gender	F	M	F	M	M	M	M	M
Duration of DM (years)	6	8	8	18	17	3	7	9
Type of DM	T2DM	T2DM	T2DM	T1DM	T1DM	T2DM	T2DM	T2DM
HbAc1 (%)	5.9	8.3	7.5	8.8	7.9	6.9	7.9	7.9
Group	IVC	IVC	IVC	IVC	IVC	IVC	Non-IVC	Non-IVC
Nonclearing VH in VE	Y	Y	Y	N	Y	Y	Y	Y
TRD in VE	Y	Y	N	Y	Y	N	Y	Y
Time of incident (weeks)	9	12	9	10	14	23	21	23
PRP completion	N	N	Y	N	N	Y	N	N

*Note*. DM, diabetes mellitus; T1DM, type 1 diabetes mellitus; T2DM, type 2 diabetes mellitus; HbAc1, glycosylated hemoglobin; VH, vitreous hemorrhage; TRD, tractional retinal detachment; VE, vitrectomized eye; Y, yes; N, no.

## Data Availability

The data used to support the findings of this study are available from the corresponding author upon request.
